# Assessing the distribution of volatile organic compounds using land use regression in Sarnia, "Chemical Valley", Ontario, Canada

**DOI:** 10.1186/1476-069X-8-16

**Published:** 2009-04-16

**Authors:** Dominic Odwa Atari, Isaac N Luginaah

**Affiliations:** 1 Department of Geography, University of Western Ontario, London, Ontario, Canada

## Abstract

**Background:**

Land use regression (LUR) modelling is proposed as a promising approach to meet some of the challenges of assessing the intra-urban spatial variability of ambient air pollutants in urban and industrial settings. However, most of the LUR models to date have focused on nitrogen oxides and particulate matter. This study aimed at developing LUR models to predict BTEX (benzene, toluene, ethylbenzene, m/p-xylene and o-xylene) concentrations in Sarnia, 'Chemical Valley', Ontario, and model the intra-urban variability of BTEX compounds in the city for a community health study.

**Method:**

Using Organic Vapour Monitors, pollutants were monitored at 39 locations across the city of Sarnia for 2 weeks in October 2005. LUR models were developed to generate predictor variables that best estimate BTEX concentrations.

**Results:**

Industrial area, dwelling counts, and highways adequately explained most of the variability of BTEX concentrations (*R*^2^: 0.78 – 0.81). Correlations between measured BTEX compounds were high (> 0.75). Although most of the predictor variables (e.g. land use) were similar in all the models, their individual contributions to the models were different.

**Conclusion:**

Yielding potentially different health effects than nitrogen oxides and particulate matter, modelling other air pollutants is essential for a better understanding of the link between air pollution and health. The LUR models developed in these analyses will be used for estimating outdoor exposure to BTEX for a larger community health study aimed at examining the determinants of health in Sarnia.

## Background

Volatile organic compounds (VOCs) are important outdoor air toxins suspected to increase chronic health problems in exposed populations [1,2]. BTEX (benzene, toluene, ethylbenzene, (m+p) xylene and o-xylene) are some of the common VOCs found in urban and industrial areas and are classified as "hazardous air pollutants" (HAPs) because of their potential health impacts [3]. Nonetheless, the evidence as to whether HAPs influence health effects remains equivocal. For example, while Leikauf [4] argued that there is insufficient evidence indicating that ambient HAPs exposure has the potential to exacerbate health problems such as asthma, the author acknowledged that once an individual with a health outcome (e.g. asthma) is sensitized to air pollution, they are more likely to respond to remarkably low concentrations of pollution. Furthermore, although low levels of VOCs might have no significant health impacts, the interaction between VOC species and other criteria pollutants might cause adverse health outcomes. Rumchev et al. [5] studied the linkages between domestic exposure to VOCs and asthma in young children in Perth, Western Australia, and found that exposure to VOCs increased the risk of childhood asthma.

Individual species within VOCs have also been examined for their health effects. For instance, the International Agency for Research on Cancer (IARC) [6] has classified benzene as a known human carcinogen based on evidence from epidemiologic studies and animal data. These studies have shown that exposure to benzene can cause acute nonlymphocytic leukemia and other blood disorders such as preleukemia and aplastic anemia [6,7]. The US Department of Health and Human Services [8] also reported an association between occupational exposure to benzene and the occurrence of acute myelogenous leukemia. In Australia, Glass et al. [9] found an association between leukemia and cumulative benzene exposures that were considerably lower than the accepted level.

Besides benzene, other BTEX compounds are also suspected to adversely affect human health. The U.S. Department of Health and Human Services [10] suggested that exposure to high dosages of toluene may cause headaches, sleepiness, kidney damage, and could impair an individual's ability to think clearly. Additionally, Chang et al. [11] reported that toluene exposure could exacerbate hearing loss in a noisy environment in Taiwan. While studying the association between several sites of cancer and occupational exposure to toluene in Montreal, Quebec, Gerin et al. [12] observed a doubling risk of esophageal cancer in subjects exposed to medium to high levels of toluene. Conversely, other studies that examined toluene as a possible risk factor for cancer did not find any significant association between exposure to toluene and cancer. For example, Antilla et al. [13] found no increase in overall cancer risk for cancers at specific tissues associated with exposure to toluene, except for a non-significant increase in the incidence of lung cancer in Finnish workers who were exposed to toluene for more than 10 years.

The evidence on the health effects of Ethylbenzene remains uncertain. Ethylbenzene has been linked to dizziness, throat, nose and eye irritations and recent laboratory assessments have shown that long-term exposure to ethylbenzene may cause cancer [14,15]. While reviewing the literature on the effects of low-level exposure to ethylbenzene on the auditory system, Vyskocil et al. [16] reported no evidence of ethylbenzene induced hearing loss after combined exposure to ethylbenzene and noise of workers in Quebec. In addition, acute exposure to xylenes could cause respiratory and neurological health problems in humans, while chronic exposure could affect the central nervous system [17]. On the other hand, work by the U.S. Department of Health and Human Services [18] provided insufficient evidence showing that xylenes are potential human carcinogens.

Although there is an understanding of the biological plausibility linking hazardous pollutants in the ambient environment to health effects, the evidence from toxicological, occupational and epidemiological studies are still frequently in discordance. This is partly due to different methodological issues. For instance, the threshold concentrations used in animal studies are frequently above those used in epidemiologic studies [4]. Also, researchers have documented that ambient (outdoor) air pollution concentrations used in epidemiologic studies may underestimate personal exposure because people spend most of their time indoors [19-21]. Despite this recognition, the argument is that the consistent pattern of outdoor air pollution when compared to indoor air pollution [20,21] means that outdoor exposure estimates may still be useful for health studies where indoor air pollution data are unavailable. That is, outdoor air pollution estimates can be used as estimates of overall pollution pattern especially in highly polluted areas such as Sarnia where the correlation between indoor and outdoor air pollution may be high as a result of traffic and industry-related air pollution [22]. Hence, in the absence of indoor air pollution estimates, outdoor exposure patterns are sufficient for health studies [23].

The equivocal nature of the relationship between ambient air pollution and associated health effects [4,24,25] may be attributed to the challenges in the assessments of ambient air pollution for epidemiologic studies [26,27]. Recently, different approaches have been proposed and utilized in addressing the challenges of estimating personal exposure to air pollution. For instance, kriging has been used both at the national and regional scale [26], but has been criticised for its inability to capture air pollution at very short distances [28]. Other studies have used proximity analysis and community average of pollution concentrations as proxies for exposure [29-31], however these approaches have also been criticised because of their high potential for exposure misclassification [32]. Microenvironment monitoring aims to address some of the exposure assessment challenges [33], but its suitability has been hampered by high costs related to data collection especially when dealing with a large cohort [34]. Traditionally, dispersion models are also used to estimate individual level exposure because they incorporate both spatial and temporal variations without the need for additional air pollution monitoring. The biggest challenge with dispersion models lies in their expensive data demands and lack of precision in the requisite meteorological or emissions data required for making accurate predictions [35,36]. Since exposure estimation can have significant impacts on explaining relationships between exposure and health outcomes [37-39], there is a growing demand for improved and affordable ways of exposure estimation that can potentially capture the variability of air pollution for health studies in high polluted environments like Sarnia [32,40].

Land use regression (LUR) modelling is proposed as a promising alternative approach to meet some of the challenges of assessing the intra-urban spatial variability of ambient air pollutants in urban and industrial settings because it can capture localized variation in air pollution more effectively and economically than some of the conventional approaches previously discussed [32,35,37,40,41]. LUR modelling predicts outdoor ambient air pollution concentrations at given sites based on the surrounding land use, traffic, population and dwelling counts, and physical characteristics such as elevation [35]. Several researchers [26,27,35] have provided critical reviews of LUR studies and emphasized the potential role of LUR models in estimating exposure to air pollution. However, most of the LUR models to date have focused on nitrogen oxides (NO_2 _and NO_x_) and particulate matter (PM_2.5_, PM_10_). With potentially different health effects, modelling other air pollutants is essential for increasing our understanding of the link between air pollution and health. Consequently, the main objectives of this study were to: 1) develop LUR models to predict VOCs, specifically benzene, toluene, ethylbenzene, m/p-xylene, o-xylene, and total BTEX in Sarnia, and 2) determine the intra-urban variations of ambient benzene, toluene, ethylbenzene, m/p-xylene, o-xylene, and total BTEX to be used in a larger community health study.

## Methods

### Study area

**Figure 1:**
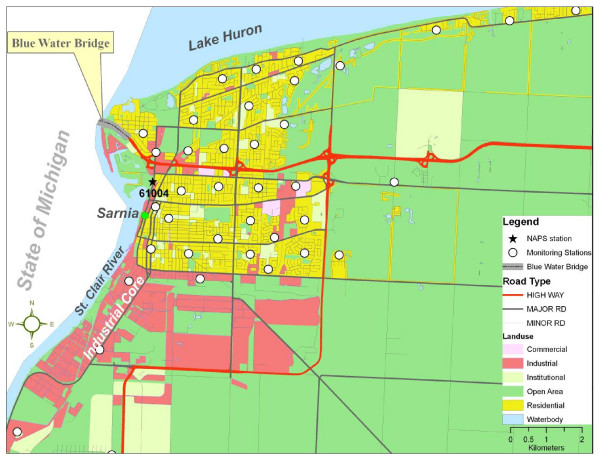
**Study area and monitoring stations**.

The City of Sarnia (42° 58' N, 82° 22' W) is located in southwestern Ontario, Canada, on the border just east of Port Huron, Michigan, USA (Figure 1). Neighbouring Canadian cities include London and Windsor. Sarnia has an approximate land area of 165 km^2 ^and a population of 71, 419 [42]. Both the city and surrounding communities are called "Chemical Valley" because more than 40% of Canadian chemicals are manufactured in this area [43]. Examples of the chemical industries in the area include Suncor, Bayer, Dow Canada, NOVA, and ESSO. Furthermore, one of the largest landfill sites in Canada known as Safety-Kleen is located in the region. These point sources in Sarnia are amongst the largest industrial polluters in Canada with the highest levels for some VOCs, such as 1–3 butadiene, compared to other polluters across the country [44]. Recently, the Canadian government designated the St. Clair region which includes Sarnia and 16 others as "Areas of Concern" based on a hypothesis that environmental pollution is negatively affecting the population in these areas [43,45].

### Data Collection – Pollution Monitoring

The BTEX species (benzene, toluene, ethylbenzene, m/p-xylene and o-xylene) were monitored using 3 M #3500 Organic Vapour Monitors (Guillevan, Montreal). Thirty-nine samplers were deployed in Sarnia for 2 weeks in October 2005 to coincide with a community health survey. The month of October best represents the average annual weather condition in Sarnia. Although formal location-allocation techniques [46] were not used, the samplers were deployed based on a number of objective criteria to capture the spatial variability of BTEX compounds in areas of high population density. Samplers were located proportional to population size in each census tract. In addition, sites were selected to ensure sufficient variability in potential predictors (e.g. land use, road networks) (Figure 1). Hence, only 2 samplers were located within Vidal Street, the main traffic route through the industrial core, which served as the point of origin for the measures for this study to capture pollution near service areas. Vidal Street is called the industrial core because it is the major traffic feeder to industries in Sarnia (Figure 1). The rest of the sampling sites were at least 600 m away from the industrial core to ensure data accurately reflected diffused ambient pollution throughout the region rather than point sources. The samplers were installed at a height of 2.5 m on light poles after obtaining permission from the City of Sarnia and the Aamjiwnaang Indian Reserve. Global positioning systems were used to geocode the monitoring locations.

The exposed filters were sent to Air Monitoring and Analysis(Mississauga, Ontario) lab for analysis of all measured BTEX species. The samples were extracted with 2.0 mL of solvent and the compounds determined using gas chromatography – mass selective detector with a detection limit of 0.1 μg/L [47]. A multi-point calibration curve (r^2 ^> = 0.99) was used and the results were corrected with lab blank, deuterated internal standard and recovery. The two-week BTEX measurements served as dependent variables in the developed LUR models.

### Assessment of spatial trends

Sampling density was calculated as the number of samplers divided by the study area. Kriging was used as the spatial interpolation technique to examine how the different BTEX species were spatially distributed based on the sampling density. The spatial trends were examined using ArcMap 9.2.

### Variable generation

The predictors of BTEX species were extracted from several datasets including traffic counts, census data, street network, land use, and digital elevation models (DEMs). The traffic counts were annual average daily traffic (AADT) volumes collected in 2004 and compiled for major and minor roads by the City of Sarnia, the Administration and Engineering Department, and for highways by the Ontario Ministry of Transportation. Both the city and provincial traffic data were then combined in GIS to establish a comprehensive dataset for traffic counts based on road segments. Population and dwelling counts at the dissemination area (DA) level were generated from 2001 census data [42]. The street network and land use 2006 datasets were obtained from Desktop Mapping Technologies Inc (DMTI) via the Data Liberation System from the University of Western Ontario. The street network file had information on all three types of roads (minor, major, highway) segment-by-segment. Digital elevation data were used to generate the elevation for each sampled station at a 25 × 25 m grid resolution (DMTI).

The independent variables were generated within circular buffers that extended from the sampling locations at 50 m intervals out to 3000 m using ArcGIS. The predictor variables were conceptually grouped into 4 different broad categories: land use, road and traffic, population and dwellings, and physical geography. The land use category included areas (in hectares) of industrial, commercial, institutional, residential, open areas and water bodies that fall within the specified buffer radii with sampling sites as centres. The roads and traffic category included calculated lengths of minor and major roads and highways; and the total vehicle miles traveled (VMT) on the roads segments that fall within the buffer radii. The VMT was calculated as AADT counts multiplied by the road segment length within a specified buffer. Calculated VMT values were then summed as the total vehicle miles traveled for the monitored station within the specified buffer. The total population and dwelling counts were calculated as the ratio of each DA that fell within a specified buffer area and the total area of that DA multiplied by total population/dwelling counts of their respective DA. Meteorological data (e.g. wind direction) was not used in the analysis because there was only one functional meteorological station in the study area during the monitoring period. The physical geography category included the x, y coordinates, elevation, measured distances from monitoring stations to Vidal Street (industrial core), the Blue Water Bridge, minor and major roads and highways.

### Model selection

The natural logarithm of BTEX species were used in the LUR modeling because their distributions were skewed. The association between the geographic variables and the mean levels of measured air pollutants was analyzed using multiple linear regression. Each of the buffers generated were individually screened through bivariate regression models using SPSS statistical software [48] to identify the variables that were highly correlated with measured BTEX species. Next, the most relevant univariate relationships were identified and then a stepwise multiple regression was conducted to find the most predictive models for benzene, toluene, ethylbenzene, m/p-xylene, o-xylene, and total BTEX (sum of all BTEX species). The final LUR models for BTEX and each species were identified as having a combination of variables with the highest coefficient of determination, *R*^2^. Independent variables retained in the models had to have significant *t*-score (*p *< 0.05) and low collinearity with other variables (defined by a variance inflation factor < 2.0).

After the most predictive models were obtained, the standard regression diagnostics to identify outliers, leverage and influence values were performed. The individual influence of each measured concentration on the whole model was examined using the size-adjusted Cook's distance [49]. Points with calculated Cook's distance values greater than the cutoff (defined as 4/sample size) were removed because of their disproportionate influence on the most predictive models. The residuals were tested for Moran *I *(MI) spatial autocorrelation [50,51]. Pearson correlations between significant independent variables in the most predictive models were also examined.

Two different cross-validation procedures to evaluate the precision of the optimized models were used. The first was a "leave-one out procedure" which involved removing one of the monitored sites and predicting the concentration at the omitted location [19,52]. This procedure was repeated for all the sampling locations and the prediction error calculated as root mean squared error (RMSE) – the square root of the sum of the squared differences of the observed and the predicted concentration at removed locations [41]. A second cross-validation approach was performed in three random selections of 90, 80 and 50% of the samplers to predict BTEX concentrations at the remaining 10, 20 and 50% locations, respectively [52,53]. The Chow test was used to determine whether the coefficients in the predictive regression models were similar to the coefficients of the three different validation trials in the second cross-validation [53,54].

The surfaces of predicted BTEX concentrations were created by applying the coefficients of the predictive model equation and generating predicted surfaces with a 5 × 5 m resolution. The correlation between kriged and LUR modeled BTEX concentrations were calculated for each sampling site. All data management and statistical analyses were performed using SPSS statistical software [48]. Spatial autocorrelation and surface generations were performed using ArcGIS 9.2.

## Results

Two of the samplers were lost due to vandalism. The two samplers were 600 and 2800 m away from the industrial core and 8200 m apart. The calculated sampling density of 0.24 was higher than for other Canadian studies in Hamilton (0.08), Toronto (0.16) and Montreal (0.18) [32,53,55,56]. With the general distribution and sampling density, the two lost samplers would likely have no significant effect on the different BTEX models. Table 1 presents the summary statistics of the BTEX compounds from the remaining 37 locations. Arithmetic means of the compounds were 0.93 ± 0.56 μg/m^3 ^for benzene, 2.58 ± 1.35 μg/m^3 ^for toluene, 0.46 ± 0.23 μg/m^3 ^for ethylbenzene, 1.21 ± 0.61 μg/m^3 ^for (m+p) xylene, and 0.49 ± 0.25 μg/m^3 ^for o-xylene. Toluene was the most abundant compound at all sampling sites followed by benzene.

**Table 1:** Distribution of BTEX concentrations at measured sites

					Percentiles		
		
	Mean	SD	Min	Max	25^th^	50^th^	75^th^
Benzene	0.93	0.56	0.28	3.36	0.56	0.86	1.15

Toluene	2.58	1.35	0.85	6.88	1.67	2.20	3.42

Ethylbenzene	0.46	0.23	0.15	1.06	0.30	0.39	0.58

(M+P) xylene	1.21	0.61	0.40	2.81	0.78	1.08	1.56

O-xylene	0.49	0.25	0.15	1.19	0.32	0.43	0.63

Total BTEX	5.67	2.88	1.83	14.50	3.69	4.91	7.36

Table 2 compares monthly (there were only 4 measurements for the month of October 2005: 1^st ^(Saturday), 7^th ^(Friday), 19^th ^(Wednesday), and 25^th ^(Tuesday)) and 5-year (2001 – 2005) means of BTEX concentrations measured at the National Air Pollution Surveillance (NAPS) station (#61004). The average ambient concentrations of the 3 sampling points closest to the station (Figure 1) were chosen for comparison following Atari et al. [55] and Miller et al. [47]. In general, the 2-week average concentrations of benzene (1.07 μg/m^3^), toluene (3.35 μg/m^3^), ethylbenzene (0.56 μg/m^3^), and total BTEX (7.19 μg/m^3^) at the 3 sampling points closest to the station were slightly lower than the monthly and 5-year means measured at the NAPS station (Table 2). The 2-week average concentrations of (m+p) xylene (1.43 μg/m^3^) and o-xylene (0.58 μg/m^3^) measured at the 3 sampling points closest to the station were slightly higher than the monthly and 5-year means measured at the NAPS station. The differences could be attributed to the fact that (m+p) xylene and o-xylene are more photochemically reactive than their counter parts [57], and different measuring instruments were used. Environment Canada used 6 Litre Summa canisters at the NAPS stations [58] while 3 M samplers were used in this study.

**Table 2:** Comparison between NAPS and sampled BTEX data

	NAPS Data^a^		Sampling Data^b^
	
	Monthly average^c^	5 years average^d^	2-week average
Benzene	1.93	1.40	1.07

Toluene	3.58	3.80	3.35

Ethylbenzene	0.64	0.57	0.56

(M+P) xylene	1.19	1.25	1.43

O-xylene	0.37	0.40	0.58

Total BTEX	7.71	7.25	7.19

^a ^Measured data at the National Air Pollution Surveillance (NAPS);

^b ^2-week average data at 3 sampling points closest to the NAPS station;

^c ^The monthly average is the mean of the only 4 records available for October 2005;

^d ^5 years average (2001 – 2005) concentration

**Figure 2:**
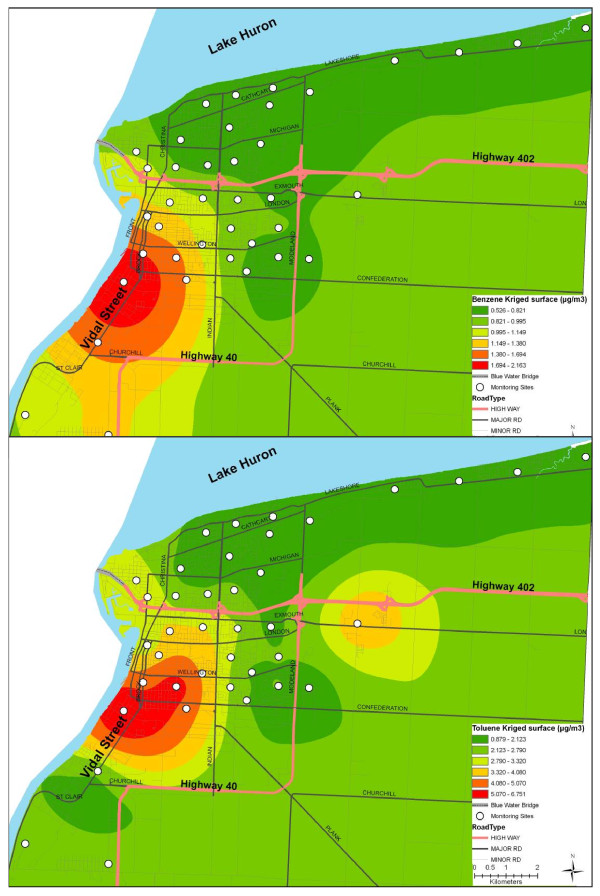
**Kriged surfaces for measured benzene and toluene**.

The measured BTEX species are highly correlated to each other (Table 3). The kriged surfaces of measured BTEX concentrations showed similar patterns with high concentrations along the industrial core. Because of the high correlation between BTEX species and their similar patterns in the kriged surfaces, only two surfaces are shown (Figure 2). The benzene surface has a slightly more localized pattern when compared to the other BTEX species. Table 4 shows the Pearson correlation coefficients between measured, kriged and LUR modelled concentrations at the sampling locations. The correlation between measured and kriged concentrations were low for ethylbenzene (r = 0.38), (m+p) xylene (r = 0.16) and o-xylene (0.14). Likewise, the correlation between kriged and LUR modelled concentrations at the sampling locations were low for ethylbenzene (r = 0.46), (m+p) xylene (r = 0.31) and o-xylene (r = -0.19). Kriged o-xylene concentrations were consistently lower than the LUR modelled concentrations at the sampling locations.

**Table 3:** Pearson correlation between measured ambient BTEX compounds

	Toluene	Ethylbenzene	(M+P) xylene	O-Xylene	Total BTEX
Benzene	0.817**	0.845**	0.752**	0.755**	0.865**

Toluene		0.973**	0.965**	0.969**	0.991**

Ethylbenzene			0.977**	0.973**	0.989**

(M+P) xylene				0.994**	0.966**

O-Xylene					0.970**

** Significant at 95%

**Table 4:** Correlations between measured, kriged and LUR modelled concentrations at sampling locations in Sarnia

	Measured	LUR	Measured	LUR
	Benzene		Toluene	

LUR	0.793**		0.719**	

Kriged	0.927**	0.828**	0.999**	0.736**

	Ethylbenzene		(M+P) Xylene	

LUR	0.384**		0.162	

Kriged	0.892**	0.458**	0.713**	0.307

	O-Xylene		BTEX	

LUR	0.135		0.614**	

Kriged	0.711**	-0.186	0.988**	0.658**

** Significant at 95%

The calculated Moran's indices for benzene (MI = -0.02), toluene (MI = 0.01), ethylbenzene (MI = -0.04), (m+p) xylene (MI = -0.03), o-xylene (MI = -0.03), and total BTEX (MI = -0.03) residuals of the most predictive models indicate no significant autocorrelation. Table 5 shows the final LUR models for predicting the concentrations of benzene, toluene, ethylbenzene, (m+p) xylene, o-xylene, and total BTEX. The model for benzene (*R*^2 ^= 0.78) included industrial land use within 1600 m, dwelling counts within 1200 m, and length of highway within 800 m. The model for toluene had an *R*^2 ^of 0.81 including industrial land use within 2800 m, open area within 600 m, and length of highway within 800 m as significant predictors. The model for ethylbenzene (*R*^2 ^= 0.81) included industrial land within 2600 m, dwelling counts within 1400 m, and length of highway within 800 m. The model for (m+p) xylene and o-xylene had similar *R*^2 ^of 0.80 including industrial land use within 1600 m, dwelling counts within 1200 m, and length of highway within 800 m. The total BTEX model had a coefficient of determination (*R*^2^) of 0.81 including industrial land use within 2500 m, dwelling counts within 1400 m, and length of highway within 900 m showing significant contribution to the model. The positive regression coefficients indicate that concentrations of BTEX compounds increase as the values of the independent variables (e.g. industrial area) rise, while the negative coefficients indicate a decrease in concentrations as the values of the predictor variables (e.g. area of open space) increase. All variables in the six models are significant at the 95% level of confidence. None of the variables in the final models were significantly correlated with each other (Table 6).

**Table 5:** Land use regression model results for BTEX compounds

Variables	Benzene	Toluene	Ethylbenzene	(M+P) xylene	O – xylene	BTEX
Intercept	-1.086 ± 0.141	0.486 ± 0.092, 5.258**	-1.879 ± 0.127, -14.758**	-1.079 ± 0.127, -8529**	-2.009 ± 0.144, -13.996**	0.587 ± 0.119, 4.927**

Industry 1600 m	0.005 ± 0.001 (0.640), 9.409**	---	---	0.003 ± 0.000 (0.339), 6.887**	0.003 ± 0.001 (0.526), 5.929 **	---

Industry 2500 m	---	---	---	---	---	0.002 ± 0.001 (0.464), 9.095**

Industry 2600 m	---	---	0.002 ± 0.000 (0.570), 10.084 **	---	---	---

Industry 2800 m	---	0.002 ± 0.000 (0.558), 8.181 **	---	---	---	---

Open 600 m	---	-0.007 ± 0.002 (0.119), -3.444 **	---	---	---	---

Dwelling 1200 m	0.002 ± 0.001(0.066), 3.153 **	---	---	0.004 ± 0.001 (0.398),7.379 **	0.003 ± 0.001 (0.219), 5.724 **	---

Dwelling 1400 m	---	---	0.002 ± 0.001 (0.157), 4.081 **	---	---	0.003 ± 0.000 (0.224), 4.997**

Highway 800 m	0.076 ± 0.024(0.073), 3.162 **	0.094 ± 0.021 (0.134), 4.543 **	0.079 ± 0.022 (0.081), 3.611**	0.062 ± 0.021 (0.060), 2.926**	0.062 ± 0.022 (0.056), 2.889 **	---

Highway 900 m	---	---	---	---	---	0.079 ± 0.018 (0.126), 4.418**

Model *R*^2^	0.779(0.757)	0.811 (0.792)	0.808 (0.790)	0.797 (0.776)	0.800 (0.780)	0.813 (0.794)^b^

Average *VIF*^c^	1.01	1.13	1.07	1.01	1.13	1.05

Model validation						

*R*^2^	0.75 – 0.81	0.77 – 0.86	0.79 – 0.86	0.78 – 0.81	0.77 – 0.79	0.80 – 0.84

RMSE^d^	0.25 – 0.87 μg/m^3^	0.16 – 0.55 μg/m^3^	0.14 – 0.17 μg/m^3^	0.27 – 0.42 μg/m^3^	0.07 – 0.21 μg/m^3^	0.58 – 1.48 μg/m^3^

**Note: **^a ^C = coefficient, SE = Standard Error, Cont = *R*^2 ^contribution; ^b ^Numbers in brackets are adjusted *R*^2^; ^c ^*VIF *= Variance Inflation Factor;

** Significant at 95%; ^c ^RMSE = Root mean square error

**Table 6:** Pearson correlation between significant variables in the most predictive LUR models

Benzene		
	Dwelling counts within 1200 m	Length of highway within 800 m

Industrial Land Use within 1600 m	0.028	-0.058

Dwelling counts within 1200 m		-0.056

Toluene		

	Open area within 600 m	Length of highway within 800 m

Industrial Land Use within 2800 m	-0.322	-0.164

Open area within 600 m		-0.107

Ethylbenzene		

	Dwelling counts within 1400 m	Length of highway within 800 m

Industrial Land Use within 2600 m	0.014	-0.205

Dwelling counts within 1400 m		0.221

(M+P) Xylene		
	Dwelling counts within 1200 m	Length of highway within 800 m

Industrial Land Use within 1600 m	0.048	-0.119

Dwelling counts within 1200 m		0.042

O-Xylene		

	Dwelling counts within 1200 m	Length of highway within 800 m

Industrial Land Use within 1600 m	0.042	-0.095

Dwelling counts within 1200 m		-0.034

BTEX		
	Industrial land use	Length of highway within 900 m

Dwelling counts within 1400 m	0.010	-0.179

Industrial Land Use within 2500 m		0.176

**Figure 3:**
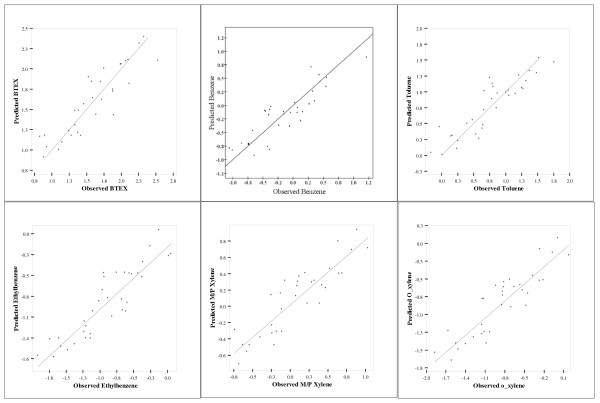
**Observed versus predicted BTEX, benzene, toluene, ethylbenzene, m/p xylene and o-xylene (logarithmic scale) based on the best land use regression models**.

**Figure 4:**
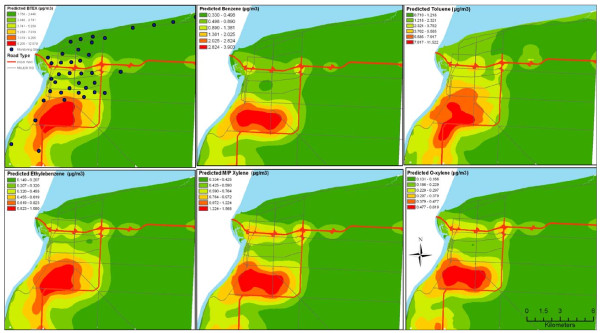
**Land use regression predicted surfaces for BTEX compounds**.

Figure 3 shows the relationship between the observed and predicted pollutants based on their natural logarithmic scales. The scatterplots reflect the strength of each of the developed models and demonstrate that the models fit the observations well with no significant outliers. The spatial pattern of the predicted BTEX species concentrations showed expected characteristics (Figure 4) compared to their kriged surfaces. The predicted surfaces reflected the significant variables with industrial area, dwelling counts and traffic showing significance. The numerous petrochemical industries along the industrial core and dwelling counts showed significant influences on the modelled surfaces. The predicted surfaces have more detailed variability compared to the kriged surfaces of measured concentrations.

The results of the validation approaches are provided in Table 5. The BTEX root mean square error predicted in this study were somewhat lower than the average estimated error of 1.72 – 2.15 μg/m^3 ^for BTEX concentrations reported by Aquilera et al. [19] who used similar approaches for cross-validation. Overall, the predicted benzene, toluene, ethylbenzene, (m+p) xylene, o-xylene, and total BTEX concentrations correspond nicely with measured concentration suggesting that these models are capable of predicting reliable concentrations. The Chow test results were not significantly different between the predictive models and the three different tests suggesting that the benzene, toluene, ethylbenzene, (m+p) xylene, o-xylene and total BTEX models developed were quite stable.

## Discussion

The aim of this study was to model the intra-urban variations of ambient VOCs including benzene, toluene, ethylbenzene, (m+p) xylene, o-xylene, and total BTEX for use in a large health study aimed at examining the determinants of health in sentinel high exposure environments. Although most of the significant variables were similar in the six models, their individual contributions to the models were significantly different. For example, while industrial land use within 1600 m was significant in both (m+p) xylenes and o-xylene models, the effect of industry (34% and 53%, respectively) differed in the two models (Table 5). These differential influences support the need for modelling the different air pollutants [55].

When compared to other LUR models developed in Munich [59], El Paso [60], Sabadell [19] and Windsor, Ontario [61], the significant variables in the present study showed considerably larger buffer radii. For example, Wheeler et al. [61] reported significant highway buffer radii of 50 m and 100 m for benzene and toluene models, respectively. In this study, we found significant highway buffer radii of 800 m for both benzene and toluene models (Table 5). The later result was also larger than the 300 m buffer radius reported by Beckerman et al. [62] when examining the variability of traffic-related pollutants around an expressway in Toronto, Ontario. The differences could be due to the unusually large number of petrochemical facilities in Chemical Valley, hence the broader distribution of ambient air pollutants in the area. The larger buffer radii found in this study potentially limits the generalizablility and transferability of the developed LUR models to areas of similar contextual and compositional characteristics [26].

When compared to other models developed in Sabadell [19], Munich [59], and Windsor, Ontario [61], the results of the various models of BTEX species are considerably different, further suggesting the need to model air pollutants in their various contexts rather than depending on proxies [37,55]. The benzene model (*R*^2 ^= 0.78) showed comparable coefficient of determination when compared to a similar model developed in Munich, Germany (*R*^2 ^= 0.80) [59] but slightly higher than the *R*^2 ^of a model developed in Windsor, Ontario, Canada (*R*^2 ^= 0.73) [61]. The toluene model showed high coefficient of determination (*R*^2 ^= 0.81) compared to similar models developed in Windsor (*R*^2 ^= 0.46) [61] and Munich (*R*^2 ^= 0.76) [59], while the coefficient of ethylbenzene (*R*^2 ^= 0.81) was comparable to the coefficient reported in Munich (*R*^2 ^= 0.79) [59]. The BTEX model developed in this study showed high coefficient of determination (*R*^2 ^= 0.81) as compared to an *R*^2 ^of 0.74 reported by Aquilera et al. [19] in Sabadell, Spain. Differences in the *R*^2 ^could be due the contextual factors in the various cities. Although the industrial area exhibited varying influences in each of the models (Table 5), the results support the view that the numerous petrochemical industries are significantly affecting the VOC concentrations in Sarnia, Chemical Valley. If possible, it is important to model each air pollutant of interest to better analyse, determine, and understand personal exposures for health studies.

Besides industrial area, dwelling counts also emerged as a strong determinant of the intra-urban variation of BTEX concentration in Sarnia (Table 5). These results are consistent with other researchers [46] who found dwelling counts to influence the intra-urban variation of air pollution. The view is that high dwelling counts may influence heavy traffic and emissions [63]. The results also indicate that a combination of land use and dwelling counts could be used to estimate exposure to air pollution, especially BTEX compounds.

The correlations between BTEX species in this study showed slightly different coefficient ranges compared to other studies in Canada and the US [62,64]. This research has slightly narrow coefficient ranges (0.76 – 0.99) (Table 3) compared to the coefficient ranges (0.53 – 0.89) reported in Toronto, Canada [62]. The difference could be due to the numerous petrochemical industries in the region. While examining the concentration and co-occurrence of VOCs in the US, Pankow et al. [64] reported comparable correlation ranges (0.78 – 0.99) between BTEX species. The high correlation coefficients in this study suggest that BTEX species are emitted by similar sources and it might be possible to monitor only one or two of BTEX species in Sarnia [47].

When compared to the measured concentrations (Table 4), kriging showed higher correlation coefficients (0.71 – 0.99) compared to the LUR modelled concentrations (0.14 – 0.79) for BTEX and all its individual components. The LUR models showed high correlations with measured concentrations for benzene (r = 0.79), toluene (r = 0.72), and BTEX (r = 0.61) but considerably lower correlation coefficients for ethylbenzene (r = 0.38), (m+p) xylene (0.16) and o-xylene (r = 0.14). When the kriged concentrations were compared to the LUR modelled concentrations at the monitoring sites, benzene (r = 0.83), toluene (r = 0.73), ethylbenzene (r = 0.51), and BTEX (r = 0.66) showed significantly higher correlations compared to (m+p) xylene (r = 0.31) and o-xylene (r = -0.19). The LUR models underestimated o-xylene concentration at the sampling locations compared to kriging. The correlation results suggest that LUR modelling could be an efficient interpolator for benzene, toluene, and ethylbenzene but not for xylenes in a highly polluted area like Sarnia. The effectiveness of kriging in Sarnia may be due to the uniqueness of the area. As mentioned, Sarnia is a relatively small region with about 40% of Canada's chemicals manufactured in the region [43].

Similar to other LUR studies, the benzene, toluene, ethylbenzene, (m+p) xylene, o-xylene, and total BTEX models were developed based on a two-week monitoring campaign. The high network deployment, monitoring, and chemical analysis cost did not permit an extensive monitoring campaign. In spite of the short-term monitoring, the models developed captured the intra-urban variability of total BTEX and its associated species in Chemical Valley. When compared, the 2-week measured concentrations at the 3 sampling locations closest to the National Air Pollution Surveillance (NAPS) station had comparable patterns with the monthly and 5-year average concentrations at the station suggesting that the measured ambient BTEX concentrations in this study were reliable. Hence, although seasonal variations may affect the temporal trend of modelled air pollution concentration, seasonality would have little influence on the spatial and geographic patterns of pollution because of the numerous petrochemical facilities in the region [53,55,63,65]. Subsequently, seasonal variation may not greatly influence chronic health outcomes because, as observed in this research, the 2-week concentrations adequately represent mean annual concentration in Sarnia (see also Lebret et al. [65])

## Conclusion

Despite the potential limitations of this research, including the short-term monitoring campaign, the development of LUR models is a relatively affordable approach that clearly offers an advantage over traditional exposure estimation methods such as dispersion models [35]. From the models developed, it is evident that in addition to industrial emissions, traffic related VOC pollutions cannot be ignored in Chemical Valley and in similar industrial areas. Because of their prevalence and potential to cause adverse health outcomes, it is crucial to model VOCs such as BTEX for increasing the research communities understanding of the link between air pollution and health. The modeled ambient air pollution surfaces generated in this study suggest that some residents may be disproportionally exposed to high air pollutants. The results suggest the need for environmental policies that help reduce industrial pollution and assist residents to reduce and cope with daily industrial exposures. The LUR modelling of benzene, toluene, ethylbenzene, (m+p) xylene, o-xylene, and total BTEX models are used to estimate personal exposure for a large community health study aimed at examining the determinants of health in a government labelled area of concern.

## Abbreviations

AADT: Annual average daily traffic; BTEX: Benzene, toluene, ethylbenzene, m/p-xylene and o-xylene; DA: Dissemination area; DEMs: Digital elevation models; DMTI: Desktop Mapping Technologies Inc; GIS: Geographic Information Systems; HAP(s): Hazardous air pollutant(s); IARC: International Agency for Research on Cancer; LUR: Land use regression; MI: Moran *I*; NAPS: National Air Pollution Surveillance; NO_2_: Nitrogen dioxide; PM_2.5_: Fine particles (particles with diameter less than 2.5 μm); PM_10_: Particulate matter (particles with diameter less than 10 μm); RMSE: Root mean square error; VOC(s): Volatile organic compound(s); VMT: vehicle miles traveled.

## Competing interests

The authors declare that they have no competing interests.

## Authors' contributions

DA and IL conceived the study and were involved in the preparation of the manuscript. DA was involved in the analysis and interpretation of the results and preparation of the paper. All authors have read and approved the final manuscript.
